# 2171: Satisfaction and perceptions of research participants in Clinical and Translational Studies

**DOI:** 10.1017/cts.2017.190

**Published:** 2018-05-10

**Authors:** Jane A. Otado, John kwagyan, Debra Ordor, Sarah Vittone, Priscilla Adler

**Affiliations:** Georgetown -- Howard Universities, Washington, DC, USA

## Abstract

OBJECTIVES/SPECIFIC AIMS: The objectives of this study were (1) to examine research participant levels of satisfaction, experiences, and perceptions; and (2) to determine best practices for researchers for engaging research volunteers in clinical trials, and thereby reducing barriers to participation. METHODS/STUDY POPULATION: A self-administered IRB approved survey on satisfaction and perceptions of research participants in clinical and translational studies was developed. The study questions were validated by 5 key informants from each of the 3 research centers who were asked to provide constructive feedback on the clarity and relevance of the questions. The final survey was a 25-item questionnaire that used a Likert scale and focused on 5 domains to reflect satisfaction with “Staff delivery of care,” “Environment,” “Center Operations,” “Study specific questions,” and “overall experiences.” Questions to reflect participant perceptions were open ended. A convenience sample of all participants currently enrolled in research studies at CTSA institutions (GU, HU, and MHRI) was obtained. In total, 131 participants completed the survey. Of these, 15 were “surrogate” partners. RESULTS/ANTICIPATED RESULTS: Eighty-two (60%) of the participants were African Americans, 40 (29%) were Whites; 94 (67%) were first time study participants. Over 90% of those surveyed strongly agreed that they were “treated well,” that their “privacy was respected,” and that they “felt comfortable asking questions of the staff.” Eighty-four percent indicated they would participate in future studies while over 91% indicated they would recommend a family member or a friend. Only 46% of participants coming for their first research visit strongly agreed that the “compensation received was satisfactory.” However, 74% of participants returning for follow-up or who had been enrolled in a previous study felt the compensation was appropriate. Seventy-four percent of those enrolled for the first time indicated “knowing the duration of this study” as compared with only 38% of repeat visitors. When asked what they liked most about participating in a research study their primary responses were “contribution to science” and “knowledge about their diseases.” Conversely, when asked what they liked least about the study they responded that the blood draws were uncomfortable and there were often barriers to transportation and parking. DISCUSSION/SIGNIFICANCE OF IMPACT: The results of this survey demonstrated that the majority of research participants rate their experience as highly favorable even among those who had never participated in clinical research previously. In some existing literature, it has been reported that financial compensation was a major motivation to participation in studies involving healthy volunteers. In this current study, however, financial compensation did not appear to be the primary motivation for participation. The participants’ at all 3 sites stated that the main reason for their participation was the increased knowledge about their disease and the contribution to science. Negative experiences cited were primarily discomfort with blood draw, transportation, and parking logistics. Most importantly, a majority of the participants stated they would participate in future studies and would recommend a family member or a friend for a clinical study. In our sample, there was no difference in the favorable ratings as determined by race/ethnicity. In conclusion, the findings of this study inform the community with regard to how the research participants rate their experiences, and thus motivate others to participate in clinical research. Reasons for participants to withdraw from trials may be associated to their dissatisfaction with a trial or with the study staff. Thus, the degree of satisfaction with the research staff and the trial itself is crucial to reducing drop-out rates and increasing compliance with study procedures. Hence participant satisfaction is key to increasing participation in clinical trials, particularly among African Americans and other racial and ethnic minorities.

